# Editorial and Miscellaneous

**Published:** 1863-05

**Authors:** 


### EDITORIAL AND MISCELLANEOUS.

*The American Medical Association.*—In the March number of the Chicago MEDICAL JOURNAL, Prof. D. Brainard, over his initials, gave certain reasons why, in his opinion, the meeting of the American Medical Association, called by the Chairman of the Committee of Arrangements, for the first Tuesday of the ensuing June, in this city, ought not to take place. Those reasons were prominently as follows:

The call emanated from but a fraction (if, indeed, from more than one member) of the Committee of Arrangements, against the known views of the remainder of the Committee.

There had been no generally expressed wish of the profession throughout the country for its re-assemblage the present year. The causes which, for the two previous years, had been deemed amply sufficient for the postponement were, and are, still present.

The time appointed is unfortunate, because on the same day the great Canal Convention is to convene in this city.

In the February number of the journal which he controls, the Chairman of the Committee uses the following language:

"Let no one outside of Chicago imagine that the course taken by the Chicago *Medical Journal* and its senior editor, Professor D. BRAINARD, in opposing the meeting of the Association, indicates any division of sentiment, or action in the Profession here, or that it represents the wishes or feelings of any one here but himself. On the contrary, the Profession here are united, and earnestly preparing to give their brethren as cordial and pleasant a reception as they have met with in any other city in our country. Our hotels are of the best character, and amply sufficient to accommodate half a dozen "Canal Conventions" and medical Associations at the same time."

The Chairman of the Committee does not attempt to controvert any of the positions taken by Prof. Brainard with the exception of the last—for the simple reason that they are wholly incontrovertible. The call was unauthorized, and if any sustain the Chairman in the course he has thought proper to take now, it is only because they believe that the mischief is already done, and all that remains is "to make the best of it." But there is a difference of opinion from which there is no escape. The large majority of the profession are totally opposed, for patriotic and practical reasons, to the convening of the Association at this time.

With regard to the hotels and the Canal Convention—although Chicago has numerous, and some magnificent, hotels, it is well known to our public, that these hotels, without any extraordinary gathering, are even now taxed to the verge of their capacity by their daily arrivals alone.

It is believed by our citizens, that this one only of the "half dozen Canal Conventions" will bring to the city a larger concourse than it has ever before known. The private residences of the city must be turned into caravanseries to accommodate the throng. The hotels will not be able to shelter a tithe of the visitors.

It may be remarked, that at the Canal Convention will be present a large number of the most prominent men in civil life—many of the most eloquent orators in the nation are to speak; and he knows but little of human nature, who thinks that any attractions for attendance which the Association can present will outweigh those which will call away very many who come to Chicago nominally to be present at its meetings.

---

*The Nitrogen of Food.*—Profs. Bischoff, Fehling and Pettenkofer, of Munich, have latterly been engaged in an elaborate series of experiments with regard to the changes undergone by food in digestion. The composition of the food given being first accurately ascertained and then the entire products of respiration and excretion carefully collected and analyzed, the result was a general confirmation of the following views:

1st. That the nitrogenous portions of the food went to repair the continuous waste of the nitrogenous tissue of the body which takes place during the performance of its various functions; and that urea was the form under which the metamorphosed or worn-out nitrogenous tissues were chiefly eliminated from the system.

2d. That when an excess of nitrogenous matter was supplied in the food, more than was sufficient to repair the waste of tissue, that excess had the effect of producing a more rapid formation and subsequent breaking up of tissue, and consequently increasing the amount of urea eliminated in a given time; but that the increased quantity of urea occuring in the urinary secretion was not due to the excess of nitrogenous matter being converted directly into that substance, as some have suppossd, but that its constituents first formed a portton of the tissues which, by their subsequent metamorphosis, furnished urea.

3d. Their experiments also showed that little or no nitrogen passed off with the fæces, or with the product of respiration, but that almost the whole evolved was contained in the urinary secretion.

---

*Precocious Pregnancy and Parturition.*—From the Registration Report to the Mass. Legislature, 1858, by Dr. Josiah Curtis, it appears that a girl of ten years, eight months and seven days was delivered of a healthy child at the full time of pregnancy. She became pregnant twenty-four days before she was ten years old. The reputed father was about fifteen years of age. The dates are accurately fixed by the official record of the town of Taunton, Mass., and by the direct testimony of Dr. Alfred Baylies of that town, who officiated both at the birth of mother and that of the child. Both mother and child are at the present time in good health and thriving. This is probably the *earliest* specimen of fecundity of which there is any reliable record.

---

*Thoracentesis.*—Cases are accumulating proving the great advantage to be derived from early employment of thoracentesis in cases of pleural effusion. N. W. Greene, M. D., of Pittsfield, Mass., in a report of several cases operated upon, remarks that his rule is, "whenever a case of pleuritic effusion does not yield readily and rapidly to medical treatment, operate." He has never seen any cause for regret, except in some cases, that it was not done earlier. As before intimated in this Journal, we endorse the rule in its broadest extent.

---

*Facial Neuralgia.*—Prompt and effectual relief is said to be often afforded by exhibition of the following mixture:

℞ Ammoniæ Hydrochlor, ʒj; Ext. Belladonna, gr. j; Syrup. Simp. f ℥ij. M.—S. A teaspoonful every fifteen minutes during the paroxysm, until the pain diminishes, which is usually after four or five doses.—*Dental Cosmos.*

---

*Tooth Powder.*—We find the following formula in the same journal:

℞ Pulv. Peruvian Bark, ℔ 1; Castile Soap, Cinnamon, *aa* ℔ 1½; Orris, ℔ 1¼; Chalk, White Sugar, ℔ 2½; Cuttle-Fish (or Pumice), ℔ 2; Oil of Wintergreen, f ℥j. Thoroughly mix in impalpable power. It is recommended to patients to use this at least once a day and brush the teeth longitudinally as well as crosswise, taking care that the grinding and lingual surfaces be not neglected. The suds formed in the mouth should be passed back and forth between the teeth, to bring it in contact with any acid which may exist there.

---

*Ovariotomy.*—Prof. E. R. Peaslee in a case of double Ovariotomy (Aug. 1862) made use, commencing upon the nineteenth day after the operation, when typhoid symptoms were imminent, of free injections into the peritoneal cavity, feeling sure that the symptoms were due to the presence of decomposing fluids. He first injected a quart of artificial serum, made by dissolving albumen with a little common salt in water, at the temperature of 98° (Fahren.) A syringe, fastened to a flexible tube, so that on removing the syringe and depressing the external portion of the tube a syphon would be formed through which the fluid would drain off. The injections were repeated from one to three times daily for the next *fifty-eight days.* The twenty-seventh day he substituted for the albuminous solution: ℞ Liqr. Sodæ Chlorinatæ, ss ℥ j; Sodii Chlorid, ℥ j; Aquae, Oj. M. The change proved highly advantageous. Prof. P. attributes the favorable ultimate result of the case to the persistent use of the injections.

---

*Ovarian Dropsy—Iodine Injections.*—D. G. Thomas, M. D., of Utica, N. Y., reports to the *Am. Jour.* a successful case of treatment of Ovarian Dropsy by Iodine injections into the cyst. Four ounces of fluid at blood heat, containing 16 grs. Iodine and 60 of Iod. of Pot., were thrown in from a glass syringe, care being taken that it should be brought in contact with every part of the sac, and it was then withdrawn by the syringe. Considerable pain and faintness was experienced at first, but soon subsided. The operation was but partially successful. Four months after it was repeated. Injected 48 grs. Iodine, 180 grs. Iod. Pot. in 8 oz. of water at blood heat. Patient had taken 30 drops of McMunn's Elixir 45 minutes before the operation to dimish the force of the shock. Much less suffering resulted.

When last seen, about six months after this operation, "she called herself well, could walk with ease three or four miles. Has gained in flesh; the size of the bowels was natural, and the walls or outline of the collapsed sac could be easily traced through the abdominal parietes."

---

*Varicose Veins.*—Dr. S. P. Turner reports several cases of successful treatment of varicose veins by means of local inflammatory action produced by a paste of Tart. Antimony and Croton Oil. The cuticle is first removed by vesication with Cantharides over a varying number of points, say from six to twelve. Tartar Emetic is then rubbed with Croton Oil to the consistency of a paste and applied by means of a probe on the denuded surface. In from thirty-six to forty-eight hours the points of application will each be marked by an umbilicated pustule, which may then be treated by warm water dressings. On the occurrence of sloughing, granulation of the ulcers will speedily occur, and the cicatrization diminish the calibre of the vein. Vesication at the outset renders the process more speedy. The recumbent position is the most favorable to success.

In superficial and not very chronic cases this will prove as useful as the twisted suture or Vienna paste, whilst it possesses the advantage of being available in almost every instance of varicose enlargement of the superficial veins of the lower extremities.

---

*Gonorrhœal Rheumatism.*—In the chronic thickening, pain and stiffness which sometimes remain after gonorrhœal rheumatism, Mr. Fuller recommends, in otherwise healthy subjects, omission of internal remedies—the cold douche to the parts daily for ten minutes or more, followed by drying and brisk friction until the warmth has returned. Then envelop the parts in lint steeped in the following lotion: ℞ Comp. Tinct. Iodine, ℥ ss; Glycerine, ℥ iiss; Aq., ℥ iij. M. The lint to be covered with flannel and kept on until the next daily douche and friction. In a few days the difficulty will generally disappear. Where the knees are affected, the structures around being thickened and effusion into the synovial cavity, he employs the same treatment, only substituting a moderately firm bandage for the flannel over the lint. Mr. Fuller thinks the ordinary method of applying the Comp. Tinct. Iodine faulty, as it can exert no distinctly absorbant influence—its local action proving a bar to its further absorption. The formula above given contains as much iodine as the skin will bear without suffering, and the glycerine saves wetting the part oftener than once in twenty-four hours.

*Burns.*—A correspondent of the London *Lancet*, recommends in burns the following preparotion: "Take chalk and linseed, or common olive oil, and mix them in such proportions as will produce a compound as thick as thin honey; then add vinegar, so as to reduce it to the thickness of treacle; apply with a soft brush or feather, and renew the application from time to time." Or, what appears to be a better plan: spread the paste over pieces of rag. These may be changed from time to time, if the pain return, exposing the parts, of course, to the air as little as possible. The writer thinks the Carbonic Acid set free by the mixture, in addition to the exclusion of the air, adds materially to the sedative effect. In the absence of oil, he suggests the whiting might be mixed with water.

---

*Medical Officers to the Prince of Wales.*—Physicians in Ordinary—Wm. Jenner, M. D., and Edward Sieveking, M. D. Surgeons in Ordinary—James Paget and George Pollock, Esqs. Surgeon Extraordinary—John Mintor, M. D., etc. Hon. Physicians—Thos. King Chambers, M. D.; Wm. Henry Acland, M. D.; and Alex. Armstrong, M. D., R. N.

In this list will be recognized names which the profession, as well as royalty, delight to honor.

---

*Operation for Hæmorrhoids.*—Henry Smith, Esq., Surgeon to King's College Hospital, has contrived a modification of Mr. Curling's clamp for holding piles during the operation of removal or the application of nitric acid. Mr. Smith substitutes in the blades a longitudinal groove and mortice, instead of the serrations used by Mr. Curling. The handles are also stiffened and controlled by small but light screws, so that the pressure may be regulated at will, thus enabling the operator to control any hæmorrhage which may occur by farther application of nitric acid, or of the actual cautery if necessary. As the operation by ligature, although usually admirable in results, is not without danger to life, this improvement may be considered a useful and important one.

*Hay Asthma.*—Ten drops of Tincture of Nux Vomica three times a day, is recommended by a recent writer, in hay asthma. The remedy may be continued for several days without apprehension or annoyance to the patient.

---

*Substitutes for Bismuth.*—The Oxychloride, Phosphate and Tannate of Tin are severally suggested as substitutes for Bismuth. The basic per nitrate of iron and the basic phosphate of iron in combination with phosphate of lime are also suggested. They may replace the Bismuth for injections, blenorrhœa and blenorrhagia, in the early stages.

It is not stated whether they will in any degree replace Bismuth for internal exhibition. We suspect not.

---

*Ext. Hœmatoxylon as a Deodorizer.*—Extract of Logwood mixed with equal parts of lard, is recommended by M. Desmartier as a deodorizer, applied to sloughing and gangrenous wounds. It, at the same time, he states, assists in restoring healthy action.

---

*Pertussis.*—Common Resin in doses, for a child, of one or two grains, and for an adult, of five or six grains, is recommended by an Australian writer as a specific for whooping cough. He often combines it with Opium or Belladonna. The remedy has the advantage of being little liable to do mischief.

---

*Administration of Quinine.*—The bitter taste of Quinine is easily concealed by putting the powder to be exhibited on a portion of the white of an egg and covering it with another portion. In this manner children, or the most "spleeny" adults will swallow it readily.

---

*Necrosis.*—Mr. Wormald, Surgeon to the St. Bartholomew's Hospital, suggests that the disintegration of dead bone is accomplished by chemical agencies. When pus is first secreted in Necrosis it is alkaline, but presently it becomes acid, and may, in some cases, be seen exuding through minute apertures which gradually enlarge, and the surface of the dead bone becomes rough. Litmus paper will detect this acid. The acid is supposed to be phosphoric—this dissolves the bone, and the air bubbles resulting may be seen on the surface of the pus. It is suggested that in doubtful cases of Necrosis, the presence of phosphoric acid may prove valuable in diagnosis; and in cases where dead bone cannot be removed by operation, Nature seems to indicate an appropriate remedy.

---

*Dropsy—Turkisk Bath.*—Dr. THUDICHUM, at a meeting of of the London Medical Society, strongly recommended the use of the Turkish bath in dropsies, where diuretics and the other usual measures fail to accomplish the desired effects. In many cases, he states, a properly constructed, well arranged, and properly heated bath of this kind was the only agent which, with certainty, would keep down the dropsy, and make the continuance of life possible. It ought always to be used in connection with the appropriate medical adjuvants. It is best adapted to cases dependent on cardiac or renal disease. When persons sweated well, the dry, hot bath for a shorter time was preferable to a longer sojourn in a lower temperature. The patients never took cold after it, and were better protected against chills than by fleecy hosiery.

---

*Syphilis.*—In the home British army one-third of the admissions into the hospitals in 1860 were on account of Syphilis, and according to the London *Times*, they exceeded one-half the average strength of the army. The disease caused a loss in the course of the year to the army at home equal to 8.69 days of the service of every soldier.

B. R. Biddle, U. S. Indian Agent in Oregon, states that with few exceptions, the Indians in his agency have the most loathsome "private diseases,"—and from this cause alone, he is led to believe that "in less than twenty-five years, these Indians will have nearly become extinct."

---

*Ice Cream Topically.*—C. P. Hart, M. D., reports to the Cincinnati *News*, a severe case of chronic dyspepsia relieved entirely by exhibition of ice cream without any other medication. The patient was ordered to take a teaspoonful of ice cream every few minutes during the day, and to allow it to dissolve slowly in the mouth before swallowing it, and to admit nothing else to the stomach while the treatment continued, either in the shape of food, drink, or medicine. In five weeks he was well, and returned gradually to his ordinary diet without any return of the gastric disorder. Dr. H. has also employed the same treatment in various inflammatory affections of the throat and stomach with the happiest results.

Certainly a combination of the *utile cum dulce.*

---

*Dyspepsia—Rennet Wine.*—The ordinary pepsine of the shops is wholly inert. If it is desired to administer this agent it may readily be prepared by taking the stomach of a calf, fresh from the butcher, "cut off about three or four inches from the upper, or cardiac extremity, which contain few glandular follicles may be thrown away. Slit up the stomach longitudinally; wipe it gently with a dry napkin, taking care to remove as little of the clean mucus as possible. Then cut it into small pieces (the smaller the better), and put all into a common wine bottle. Fill up the bottle with good sherry, and let it remain corked for three weeks; at the end of this time it is fit for use. Dose—One teaspoonful in a wine glassful of water immediately after meals."

Wouldn't a little more of the wine, without the rennet answer a better purpose?

---

*Phthisis.*—Dr. McCormac, of Belfast, insists that consumption is the sole product of respiration of air which has already been respired. The insufficient discharge of carbonaceous waste leads to its accumulation in the blood, and final deposit, in the form of tubercle, in the living tissue; and this foreign body is incompatible with the continuance of healthy life. "The one and only cause of consumption and scrofula, is breathing over and over the same breath. The one and only preventive is not to breathe afresh one's own breath or the breath of others." "Impurity of the atmosphere, say as generated by putrefaction, is not productive of consumption. It is only the atmospheric impurity which arises from the act of respiration which is so."

He believes that the ravages of consumption may be entirely put a stop to by conforming to the laws of the organism he thus has indicated.

The views of Dr. Bowditch, to which we called attention some months since, will illustrate, with these of Dr. McCormac, the errors to which the best minds are liable when they seek in single causes the result of a concurrence of causes. Dr. McCormac refers everything to the effects of repeated respiration of the same air—Dr. Bowditch to certain hygrometric conditions of the locality. It would be just as easy—easier, indeed, to refer to the kind of food taken—just as easy to refer to the condition of the digestive organs.

But the fact remains—consumption is not brought about by any single cause, but by a concurrence of causes, each of which must be thoroughly investigated in each case.

---

*Cannabis Indica—Chronic Diarrhœa.*—Dr. T. L. Wright, of Bellefontaine, Ohio, recommends, through the *Lancet and Observer*, two grain doses of Cannabis Indica in camp or chronic diarrhœa, where opium and other remedies only partially succeed. This remedy, he says, under these circumstances, he has never known fail to ameliorate the condition of the patient. He sometimes combines it with quinine, opium, or acet. plumbi. It is adapted to the later stages of the disease. Care should be exercised against its development of the *cumulative* effect. He attributes its beneficial effect to its direct strengthening influence upon the nerves.

---

*Cider in Erysipelas, &c.*—Dr. A. McBride, in the same journal, recommends good sound cider to erysipelatous patients "as often as they would drink it." He remarks that he also gives the same to his pneumonia and fever patients. Where it cannot be obtained, he recommends vinegar and water, sour wines, fruits, &c. The practice is strongly urged upon the Sanitary Commission. It is well adapted to remove the cutaneous tinge of icteric patients.

---

*Ghosts.*—The *London Lancet* says that Mr. Pepper is lecturing at the Polytechnic Institute, and displays an ingenious contrivance whereby any amount of very highly finished ghosts may be furnished to order. These are raised by the aid of a strong light, a mirror, a few lenses and some smoke.

This will recall the arrangement of the French *savant* which so startled the not over bold King Louis, and led to the disgrace of both optics and the optician at the Tuilleries. From that scrap of history, it is prognosticated, that the overthrow of the popular belief in ghosts and hobgoblins, fondly anticipated by the *Lancet*, as one of the results of Mr. Pepper's expositions, though a consummation devoutly to be wished, is not yet on the eve of accomplishment.

---

*Canada Lancet*—William Edward Bowman, M. D., Editor, Montreal.—This is a monthly journal, and is published at one dollar *per annum* in advance. All communications are to be addressed to the Editor, McGill St., Montreal. We cheerfully place this new candidate for professional favor on our exchange list, and invoke for it the liberal patronage of our Canadian neighbors. Canada ought certainly to sustain at least one journal liberally. And we beg leave to remark, right here, that by sustaining a medical journal we mean to include *the*

*use of the pen* as well as the forwarding of subscriptions. If the profession wish a live medical journal among them, they should feel an individual responsibility in furnishing original communications. We trust our new *confrere* will find an abundance of able contributors, to relieve the otherwise arduous and thankless labors of the editorial position.

---

*Inoculation in Intermittent.*—All Western practitioners have noticed on the decline or interruption of intermittents and remittents, that patients are not unfrequently affected with what are popularly designated as "cold sores," a variety of *Herpes labialis.* In "malarious districts" this is called "a receipt for the ague," as it is generally believed to indicate cure of the original affection. E. Lynch, M. D., of Lancaster, Ohio, (Philadelphia *Reporter*,) in a case of obstinate intermittent disorder, tried the experiment of inoculating with matter from some of these pustules in the usual form. Six days after the inoculation the patient had an eruption on various parts of the body, after which there was only one slight return of the intermittent symptoms. From the results in this case, Dr. L. suggests further experiments to ascertain whether by this method susceptibility to the distressing effects of "malaria" may not be eradicated.

The objection will at once occur to Western practitioners that patients who have had this labial eruption "the natural way," seem fully as liable to recurrence of the paroxysms, as do those who have escaped without this disagreeable sequel to previous paroxysms. In our experience we have been led to believe that the occurrence of the labial eruption more commonly indicates that the patient had taken an insufficient amount of Quina to arrest the intermittent. Some patients are especially liable to this eruption every time they have intermittent, and to these we have learned to administer doses of Quina much more abundant than those usually found necessary. We suggest that in Dr. Lynch's case the strong impression made upon the mind of the patient had much to do with the favorable result. It is to be regretted that the Dr. did not describe the character of the eruption which appeared on the sixth day. Was it not simple urticaria?

---

*Sub-Involution of the Uterus after Parturition.*—Dr. A. P. Dutcher (Philadelphia *Reporter*) calls attention to the increased frequency of cases of hypertrophy, or sub-involution of the uterus, after parturition or abortion. He attributes this to "our artificial state of society, and the increasing facilities for producing criminal abortion." When a woman has once miscarried or aborted, it is well known that she is very liable to the same accident, at the same period of the next pregnancy. It is also a remarkable fact, that such women often become pregnant again in a very short time after the occurrence of the miscarriage. This condition of affairs interferes materially with the proper restoration of the uterus to its normal state by involution, and thus it is apt to become permanently hypertrophied, producing the various unpleasant general and local symptoms which characterize such cases. The hypertrophy is readily to be recognized by the usual methods, provided it is present and *sought for.* The symptoms are too often mistaken for those of hysteria, and what is worse, unfortunately, sometimes for (that pack-horse loaded with female suffering) ulceration of the *os uteri.*

To promote complete involution of the organ and restoration of the normal functions, Dr. Dutcher especially recommends a combination of bi-chloride of mercury and muriate of ammonia, viz: ℞. Hydrarg. Bi-Chlorid. gr. j; Ammoniæ Hydrochlor., ʒ ij; Aq. Font., f℥ ij. M. S.—A teaspoonful three times a day in a teacupful of decoction of Scutellaria. The combination of the first two, he remarks, affords an agent of wonderful remedial power in such cases. "It acts upon the absorbents of the uterus with great energy, stimulating them to perform their functions more vigorously, and causing them to eliminate the effete matters that are engorged in the tissue of the organ, and thereby reducing it to its normal dimensions." He considers that both the Iodide and Bromide of Potassium, which some writers recommend, are quite inert when compared with this combination. The only cases in which they might be preferable are those clearly of a tuberculous tendency. Chalybeates, nervous stimulants, strychniæ, &c., may be found necessary, either contemporaneously or subsequently.

---

*Shortening after Fractures of Lower Extremities.*—J. H. H. Burge, M. D., Surgeon of the L. I. Coll. Hospital, advises, in measurements, to ascertain the degree of shortening after fractures of the lower extremities, to choose as the fixed points, the anterior superior spinous process of the ilium and the internal malleolus of the tibia. These points are so well defined at their lower border that sufficient exactness will be obtained. "All the real sources of fallacy in this method are such as can be easily overcome. They are as follows: 1st. When one limb is abducted more than the other. 2d. When the body and hips are inclined to either side, and 3d. When (as will often happen) extension and counter-extension are employed, the body and limbs cannot be brought to a straight line with each other." From the observations made and carefully tabulated, it would appear: "1st. That for every three inches an adult limb is abducted, it is shortened one-eighth of an inch; and the limb which remains on a straight line with the body, will be shortened one-fourth of an inch when the other is abducted to the extent of thirty-three inches. 2d. For every eight inches that both limbs are inclined to one side of the body, *i. e.* the one adducted and the other abducted; the adducted limb is lengthened one-eighth of an inch; and for every sixteen inches of the inclination, the abducted limb is shortened one-eighth of an inch."

In measurements, the same proportion will hold for younger persons. From the minute change, notwithstanding considerable variation from the straight line, this particular method is seen to possess great superiority to the ordinary modes adopted, and is, therefore, well worth bearing in mind.

---

*The N. Y. Ophthalmic Hospital* and School held its Eleventh Anniversary on the 24th of February in the University Medical College in 14th street, before a large and highly respectable audience. The exercises of the evening were introduced with prayer by the Rev. E. Thomson, M. D., Editor of the *Christian Advocate and Journal.*

After some introductory remarks by Solomon Jenner, A.M., President of the institution, the names of the graduating class were read by Mark Stephenson, M. D., Lecturer on the Anatomy, Pathology and Treatment of Diseases of the Eye, who remarked to the President, that these young gentlemen (seventeen in number) were students from the different Medical Colleges in this city. Previous to this unfortunate rebellion, our classes were much larger, yet never have we had one more studious or more indefatigable in their clinical pursuits at the Hospital.

He also remarked that they had attended his lectures on Ophthalmic Surgery; been examined every Saturday by Dr. M. P. Stephenson; attended the cliniques three days in the week; learned to diagnose diseases of the eye, watched the effects of remedies from day to day, and witnessed the various operations at the Ophthalmic Hospital, where over one thousand patients are treated every year.

He then said he believed they had seen more diseases of the eye and were better prepared to practice this department of their profession than many physicians who have been twenty or thirty years in practice.

He complimented the class upon their final examination before each of the attending surgeons—and further remarked they were worthy of the testimonials about to be conferred upon them. The diplomas were then presented by the President to the following gentlemen, several of whom were graduates, and had been a number of years in practice:

Alex. E. Jenner, M. D., Ohio; John P. Schenck, Jr., Dutchess Co., N. Y.; W. J. Orton, Broome Co., N. Y.; DeWitt Webb, Dutchess Co., N. Y.; J. H. McCann, M. D, Louisville, Ky.; Robert King, Geneva, N. Y.; H. G. Olmsted, M. D., N. Y. City, N. Y.; James Hutchison, St. John's, N. B.; Charles R. Sanderson, M. D., Ohio; J. H. Chittenden, Binghampton, N. Y.; James H. Mills, M. D., Orange Co., N. Y.; Geo. C. Hayunger, Canada West; J. H. Hunter, M. D., Concord, N. H.; M. C. Rowland, M. D., Washington Co., N. Y.; J. M. Waddle, M. D., Arkansas; R. J. P. Morden, M. D., Canada West; Thos. Thompson, Delaware Co., N. Y.

The graduates were then addressed in a very forcible and impressive manner, well adapted to the occasion, and replete with judicious counsel and admonition, by Marcus P. Stephenson, M. D., one of the attending surgeons, under whose immediate instruction and private examinations they had been, during the past winter. The valedictory address was delivered by A. E. Jenner, M. D., the orator of the evening, chosen from the graduating class. It was attractive in style, profound in thought, and will be long remembered by that intelligent audience, and particularly so by his fellow students.

The entertaining exercises of the evening were closed by an eloquent address from John P. Garrish, M. D.

Before the benediction was pronounced by the Rev. Dr. Thomson, the President announced that the next Session would be commenced on or about the middle of October, at the Ophthalmic Hospital, corner 4th avenue and 28th street.

---

*Camp Diarrhœa.*—Prof. Alonzo Clark reports that in his hands the per-nitrate of iron with opium has proved more successful than any other remedy in controlling and ultimately curing army diarrhœa. Opium alone has been "found wanting," and bismuth, soda and nux vomica, cod liver oil, &c., of no avail. A simple diet has usually been ordered, but a mixed, or even promiscuous diet has rarely proved particularly prejudicial. Indeed in the camps and hospitals it has usually been found that those who have the run of the cooking department soonest recover.

Dr. Clark found Bright's disease a very frequent concomitant of the diarrhœa.

We take the occasion to remark that active astringents are of little real efficacy in the vast majority of these cases. Well chosen, easily digestible articles of diet, fresh air, bathing, and tonics have succeeded best in our hands. The very elegant preparation of Bark and Iron, advertised on the cover of this journal by Mr. Sargent, has proved peculiarly efficient and satisfactory.

---

*Gonorrhœa.*—John Hastings, M. D., U. S. Marine Hospital, San Francisco, reports in the *Pacific Journal* his treatment of gonorrhœa. Patients in the acute stage are freely purged, kept quiet and placed on half-diet for three days, after which they are allowed full diet. The urethra, from the time of reception, is injected night and morning with a saturated infusion of fresh Hydrastis Canadensis. He is first directed to urinate, and after the injection to lie on his back for an hour so as to retain it. After the first purgative, no medicine is given internally. This treatment allays the chordee and ardor urinæ almost immediately, and in the course of a few days the disease is removed. Dr. H. says that, having used all kinds of treatment, he finds this produces a quicker cure, with less pain to the patient, than any other.

Dr. Hastings has also employed the infusion of Hydrastis as an injection into the bladder in Cystitis. For this purpose the temperature should be brought to blood-heat, and about four ounces thrown in daily. The pain on micturition is remarkably relieved. It, at all events, has this marked advantage, that it replaces with an innocuous agent the harsh and severe applications too frequently resorted to in such cases.

*Pain in Diseased Bladder.*—It is recommended, when the urine or calculous concretions produce great pain in the bladder, uncontrolable by the usual measures, to place the body upon an inclined plane, the hips being elevated. This throws the urine or other contents upon the upper and posterior parts of the bladder, which are much less sensitive than the *bas fond.*

---

*Diabetic Urine*, even when long exposed to the air, does not, like ordinary urine, give off any smell of decomposition. The sugar is converted into alcohol, which passes off by evaporation. This fact affords a tolerable test, when others are not at hand, for the presence or absence of sugar.

---

*Illegitimate Births Per Annum.*—In London, 4 per cent.; in Paris, 33 per cent.; in Brussels, 35 per cent.; in Munich, 48 per cent.; and in Vienna, 54 per cent. In Antigua in a single quarter the births were 194, of which 100 were illegitimate. The number of deaths during the same quarter was 460.

In this country the prevalence of the practice of procuring abortion lessens the proportion of illegitimates—to the unjust credit of its morality.

---

*Vaccination During Pregnancy.*—Prof. Meigs in his work on Obstetrics earnestly urges that under no circumstances should a pregnant woman or one recently confined be vaccinated. (*Op. citat.* p. 479.) He observes that he coincides with the almost unanimous sentiment of the profession that the woman who is confined during small pox dies. The vaccine virus, he argues, is but the variolous poison modified by passing through the system of the cow. He continues:

"The generical force of the inferior animal has modified a poison produced by the generical force of the human being. It has changed, not destroyed it. It retains a portion of its variolous power, which is inimical to the pregnant woman, and to expose one to its rage is a gross imprudence and misapprehension which I hope no student reading this book will ever be guilty of. The shocking spectacles of distress that I have witnessed, from the vaccination of pregnant females, have so impressed my mind with the enormity of the imprudence, that nothing, I think, could tempt me to commit it myself. The most furious phlebitis, which is endangitis, and which becomes pyæmic fever, is one of the consequences likely to result from every true or spurious vaccination of a pregnant woman."

This is very strong language, and the high authority from which it comes entitles it to consideration. As for ourselves, we freely confess that, entertaing the general view of the imminent danger to the mother, confined during small pox, we have always urged vaccinnation if that disease was prevalent at the time; and, although we have certainly vaccinated scores in that condition, we do not recollect a case with more than the usual unpleasant consequences. Will some of our correspondents report their experience on this point?

---

*Obstetrics: The Science and Art.*—By CHARLES D. MEIGS, M. D., lately Prof. of Midwifery and the Diseases of Women and Children in Jefferson Medical College at Philadelphia; Corresp. Memb. of the Hunterian Society, London, &c., &c. Fourth Edition, Revised. With one hundred and twenty-nine Illustrations: pp. 730. Philadalphia: Blanchard & Lea. 1863.

This is a new edition of a standard work, and we do not need to say more to our readers than that it is not merely a reprint from steretype plates, but has been thoroughly revised and improved by omissions, additions and corrections. The form has been materially changed by dividing the whole work into paragraphs, numbered from 1 to 959, thus enabling the student or practitioner to refer with ease to any topic sought. The article on placenta prævia has been entirely recast, the author wishing to notice certain new modes of treatment which he regards "as not only ill-founded as to the philosophy of the department, but dangerous to the people."

Prof. Meigs has established a reputation in his department of teaching which is world-wide. Brilliant and fascinating as a lecturer, as a writer he is scarcely less entertaining; his book is a reflex of his mind—a photograph of his opinions. He is tenacious of his opinions, sometimes, in our view, incorrect—verging upon the dogmatic, but there can never be any doubt what his opinion is, for it is brought out clear as the sunlight. We shall have occasion to comment upon certain points of his instruction hereafter which our space will not at present permit.

Taken as a whole it is an invaluable work, which should be in the library of every practitioner—to be studied for its scientific and practical precepts, and to be admired for its artistic beauty as a specimen of typographical art.

---

*On Diseases of the Skin.*—By Erasmus Wilson, F. R. S. Fifth American from the Fifth and Revised London Edition. With Plates and Illustrations on Wood; pp. 649. Philadelphia: Blanchard & Lea. 1863.

This a truly magnificent work—magnificent not from its extent, but as a monument of genius, erudition, acute observation, philosophical analysis and lucid practical description. It stands deservedly at the head of all works upon the subject of which it treats. Diseases of the Skin—from the study of which the student shrinks—for which the most enthusiastic practitioners, with very few exceptions, betray an ill-concealed repugnance, and "the very nomenclature of which," as Alibert tersely expresses it, "is a terror!"

But, in reading these elegant essays of Wilson, the beforetime, dry and tedious branch of study positively becomes a recreation and a luxury. It reminds one of Darwin, and the times when a medical man could be a *litterateur* and a *savant* without necessarily being dull and soporose. Great accuracy of perception and refinement of description do not require indispensably the husky barrenness of a botanical grouping of qualities.

The first chapter is devoted to the Anatomy and Physiology of the Skin; the second to Classification; the third to General Pathology; and the fourth to General Therapeutics. Then follows a discussion of the subject matter of the work under two grand divisions: 1st. Diseases affecting the General Structure; 2d. Diseases affecting the Special Structure.

Under the first division he enumerates: 1. Diseases arising from general causes; 2. From special external causes; 3. From special internal causes; 4. From the syphilitic poison; 5. From animal poisons of unknown origin, and giving rise to eruptive fevers.

Under the second division: 1. Diseases of the vascular structure; 2. Of the nervous structure; 3. Of the papillary structure; 4. Of the pigmentary structure; 5. Sudoriparous organs; 6. Sebiparous organs; 7. Hair-follicles and hair; 8. Nail-follicles and nails.

The classification is clear and explicit, and the simplicity of the nomenclature adopted is worthy of all praise. The author does not find it necessary to make any display of wisdom by the invention of new specific designations. Indeed we do not recollect in the whole work a single new word "of learned length and thundering sound" to give fictitious dignity to some mere variety of a well known cutaneous disease.

It is safe to say that in no part of practice are medical men so generally negligent or deficient in knowledge as in this particular branch; and yet there are scarcely any forms of disease more frequently brought to their notice than those involving the skin. In this book the practitioner will find every part arranged for ready reference, with practical directions, definite, direct, and unequivocal. The student will find a distinctness of description and graphic portraiture which will enkindle his interest, impress his understanding, and indelibly imprint the subject upon his memory.

The few pages devoted to the hair-follicles and hair, alone are worth the pecuniary cost of the whole work; and the chapter on Syphilodermata, that and a dozen times more.

Although not so immediately practical, the chapter on Elephantiasis is exceedingly interesting and will richly repay perusal.

---

*Transparent Paper.*—It occasionally happens that it is desirable to copy sketches, diagrams, etc., a task which, trivial as it may appear to an expert, is a serious enough one to those not skilled in the art. It is well to know that Benzine, applied by a sponge over the paper used for tracing the design, will render it temporarily transparent, so that all that is necessary for the copyist to do is to follow the markings on the original with pencil or India ink as desired. The benzine will speedily evaporate, leaving the paper as before opaque. The odor of the benzine will disappear in a few hours. Ordinary tracing paper, rendered transparent by oils, does not give by any means so distinct a result, neither can it be so readily colored if coloring be desired. The benzine process was discovered by Prof. Oelschlager.

---

*National Reward for Anæsthesia.*—We have received, from some unknown hand, another bulky pamphlet in the shape of a report from a Congressional Committee on the petition of W. T. G. Morton to be awarded a compensation for the use of Ether in the army. His claims are fully set forth by the Com., but they fail to make any special recommendation in the premises. We regret this, because we believe, if ever any man was entitled to reward for invention or discovery, Morton is. Some of our *confreres*, we observe, are disposed to ridicule, or at least throw cold water on the claim, mainly, so far as we can discover, because W. T. G. happens to be a dentist instead of a doctor, and is in some disrepute for something like genteel begging from distinguished medical gentlemen and medical societies and institutions.

On the whole it *does* appear that W. T. G. is anything but amiable at the reception his claim has met, and has not precisely suited in his movements the æsthetical ideas of the preservers of the dignity of the profession.

But, notwithstanding the alleged disagreeables of W. T. G. in his *personnel*, and his vain attempt to impose the burdens of a patent right upon those who employ anæsthetics, we still recall the immense boon which, whether willingly or unwillingly, has been conferred by him on the profession and the world—[and from our own immediate recollection of the facts of its introduction, we believe he was the man, and the only man, who has any shadow of claim to priority *practically*—either in its discovery or introduction]—and hence we sincerely trust that W. T. G. M. may have at least the $100,000 which his friends ask for him from Congress. This is by far too little, in our opinion, but this at least should be granted; not grudgingly, but with a free and generous sense of justice. A hundred years from now, and the whole world will wonder at the parsimony, ingratitude, and injustice which now stumbles and hesitates at this unquestionable act of common honesty.

---

*Chemistry*. By Wm. Thos. Brande, D. C. L., F. R. S. L. & E., &c., &c., and Alfred Swaine Taylor, M. D., F. R. S., &c., &c. *Experimentis et Præceptis*. Blanchard & Lea, Philadelphia, 1863.

So thoroughly are these eminent publishers advised as to the merits of the various works they reprint, that the very fact of the republication of a foreign book is about sufficient evidence of its valuable character. The present volume is altogether timely, and from a cursory glance it seems to be fully up to the standard of the science. It was received too late for particular notice the present month, but will receive due attention in the next issue, a professional friend of high distinction in this department having been requested to give the book a thorough examination.

*Camp Fever.*—Surgeon ALEX MCBRIDE, in one of his valuable communications to the *Lancet and Observer*, characterizes Camp or Scorbutic Fever as differing materially from, although in many resembling the Typhoid Fever of Louis, or Enteric Fever of Wood. He thus tabulates the

DIFFERENTIAL SIGNS AND SYMPTOMS.

| CAMP FEVER. | ENTERIC OR TYPHOID FEVER. |
|---|---|
| Abdomen soft, with little or no tenderness. | Abdomen tympanitic, with tenderness more or less intense. |
| Tongue generally large; frequently flabby and pale. | Tongue generally small, and red where not coated. |
| Tongue fissured always; fissures peculiar and continued through the entire course of disease; not dependent on dryness. | Tongue cracked when dry, but fissures not persistent; disappearing when the tongue becomes moist. |
| Appetite generally good, and solid food well borne in considerable quantity, but digestion imperfect. | Appetite slight, and food not borne except in small quantity of liquid form; digestion perfect when too much not taken. |
| No characteristic eruption during any stage of the disease. | Characteristic rose-colored eruption, usually in second or third week. |
| Delirium caused apparently by uræmic poisoning. | Delirium caused apparently by enteric inflammation and the general action of fever on sensorial system. |
| Alvine evacuations large and not characteristic. | Evacuations small and characteristic. |

It is attributable to the diet and vicissitudes of camp and general military life. Briefly: "Large quantities of agotized food, imperfectly prepared; privation of anti-scorbutic food; long continued exposure to low temperature and damp air; the widest possible range of mental and moral excitement and depression." He is of the opinion that inflammation of the intestinal glands does not belong to this disease, although there is an excited or irritated condition of the mucus membrane caused by the passage of undigested and vitiated food over it, combined with the long continued exposure to cold and damp. The alkalies, the vegetable acids, acidifying fruits and vegetables necessary to digestion and assimilation are furnished in insufficient amount, and hence the scorbutic phenomena attending the essential diarrhœa.

In the treatment, he recommends Opium, the gum, powder or tincture, combined when indicated, with tannic acid or per sulphate of iron, or the tincture of the chloride of iron. The bowels to be moved, if at all, with castor oil and laudanum.

Quinine and Camphor with or without the addition of Morphia, is frequently of happy effect. Sp. Mindereri in half drachm or drachm doses, is an excellent remedy in his experience, "to promote moisture of the mouth and skin." The dry tongue, amenable to turpentine as it is in common typhoid, receives no benefit from that agent in the camp fever. "The partial suppression of urine which is perhaps the most fatal incident in this disease," is relieved best by the acetate of potash. Nitrous ether and acid vegetable drinks may be also employed freely.

He mentions Mercury only to condemn its use *in toto*, in cases of the kind. Epispastics are very useful in complications.

But the author wisely concludes that medicines are in these cases of altogether secondary importance. Regimen is everything, or paramount to everything. Cleanliness, warmth, free ventilation. Common food, well prepared, with a preponderance of anti-scorbutic vegetables. Patients low with the disease often eat with benefit, boiled potatoes and finely cut cold slaw. Cabbage and potato soups, boiled turnip with butter, milk, sweet or sour, according to taste, &c., &c. The patient's appetite, usually good, may be considerably indulged. Hard cider, diluted native wines, or in the absence of these, diluted vinegar, sweetened. Citric or Tartaric acids may be used for variety—so also jellies, sour fruits, &c., &c. Regimen like this, he believes, will suffice for the recovery of most cases of scorbutic or camp fever.

---

☞ Business letters to the Journal should be addressed to Dr. E. INGALS, Chicago, Ills., P. O. Drawer 5787. When money is received a receipt will be returned with the next number of the Journal, and subscribers failing to get such receipt will confer a favor by giving us notice of the omission.

---

Vaccine Matter.—Fresh and reliable Vaccine Matter may be obtained by enclosing One Dollar and addressing

Chicago Medical Journal,
P. O. Drawer 5787, Chicago, Ill.

